# RUNX3 regulates the susceptibility against EGFR-targeted non-small cell lung cancer therapy using ^47^Sc-conjugated cetuximab

**DOI:** 10.1186/s12885-023-11161-1

**Published:** 2023-07-12

**Authors:** Da-Mi Kim, So-Young Lee, Jae-Cheong Lim, Eun-Ha Cho, Ul-Jae Park

**Affiliations:** Radioisotope Research Division, Korea Atomic Energy Research Institute, Daejeon, 34057 Republic of Korea

**Keywords:** EGFR-expressing NSCLC, Radioimmunotherapy, Cetuximab, Scandium-47, RUNX3

## Abstract

**Background:**

Radioimmunotherapy with cetuximab and conjugates with various radioisotopes is a feasible treatment option for different tumor models. Scandium-47 (^47^Sc), one of several β^−^-particle-emitting radioisotopes, displays favorable physical and chemical properties for conjugation to monoclonal antibodies. However, the therapeutic efficacy of ^47^Sc in preclinical and clinical studies is largely unknown. Given that intrinsic alterations in tumors greatly contribute to resistance to anti-epidermal growth factor receptor (EGFR)-targeted therapy, research on overcoming resistance to radioimmunotherapy using cetuximab is required.

**Methods:**

^47^Sc was produced by irradiation of a CaCO_3_ target at the HANARO research reactor in KAERI (Korea Atomic Energy Research Institute) and prepared by chromatographic separation of the irradiated target. Cetuximab was conjugated with ^47^Sc using the bifunctional chelating agent DTPA. Radiochemical purity was determined using instant thin-layer chromatography. The immunoreactivity of ^47^Sc-DTPA-cetuximab was evaluated using the Lindmo method and an in vitro cell-binding assay. The inhibitory effects of cetuximab and ^47^Sc-DTPA-cetuximab were confirmed using cell growth inhibition and BrdU cell proliferation assays. Differences in protein expression levels between cetuximab- and ^47^Sc-DTPA-cetuximab-treated cells were confirmed using western blotting. Complex formation between RUNX3 and DNA repair components was confirmed using immunoprecipitation and western blotting.

**Results:**

Cetuximab induces cell cycle arrest and cell death in EGFR-overexpressing NSCLC cells. Radiolabeling of cetuximab with ^47^Sc led to increased therapeutic efficacy relative to cetuximab alone. Application of ^47^Sc-DTPA-cetuximab induced DNA damage responses, and activation of RUNX3 significantly enhanced the therapeutic efficacy of ^47^Sc-DTPA-cetuximab. RUNX3 mediated susceptibility to EGFR-targeted NSCLC therapy using ^47^Sc-DTPA-cetuximab via interaction with components of the DNA damage and repair machinery.

**Conclusions:**

^47^Sc-DTPA-cetuximab promoted cell death in EGFR-overexpressing NSCLC cells by targeting EGFR and inducing DNA damage as a result of β irradiation emitted from the conjugated ^47^Sc. Activation of RUNX3 played a key role in DNA damage and repair processes in response to the ionizing radiation and inhibited cell growth, thus leading to more effective tumor suppression. RUNX3 can potentially moderate susceptibility to ^47^Sc-conjugated cetuximab by modulating DNA damage and repair process mechanisms.

**Supplementary Information:**

The online version contains supplementary material available at 10.1186/s12885-023-11161-1.

## Background

Epidermal growth factor receptor (EGFR) is an important biomarker for non-small cell lung cancer (NSCLC). Several molecular-targeted agents have been developed and studied to inhibit EGFR activity in oncology therapy [1]. Despite improvements in cancer therapy strategies, only a minority of patients have benefited from EGFR-targeted monotherapy. A combination of EGFR-targeting agents with chemotherapy or radiotherapy has been developed to improve the effectiveness of cancer treatment and its application to various patient populations. Radioimmunotherapy (RIT), a type of anti-cancer therapy, combines immunotherapy and radiotherapy, using engineered monoclonal antibodies and therapeutic radioisotopes as agents for targeted therapy and carriers of radioactivity, respectively. RIT takes advantage of the “cross-fire” effect of ionizing radiation emitted by radioisotopes conjugated with tumor-specific targeted monoclonal antibodies [2, 3]. Many preclinical and clinical studies have reported that RIT shows significantly superior therapeutic efficacy compared with immunotherapy alone using unconjugated monoclonal antibodies [4, 5].

Cetuximab, a chimeric anti-EGFR IgG1 monoclonal antibody, is currently an effective agent for the targeted therapy against head and neck squamous cell carcinoma (HNSCC) and colorectal cancer (CRC) in clinical therapeutics. Cetuximab has a high binding affinity for the extracellular domain of human EGFR and abrogates ligand-induced EGFR phosphorylation [6, 7]. It can block ligand binding, inhibit cell-cycle progression or DNA repair [8], induce the regression of angiogenesis [9], increase the internalization of receptors [10], and promote antibody-dependent cellular cytotoxicity (ADCC) [11]. However, the results obtained after using cetuximab as the sole agent for anti-EGFR-targeted therapy were not as beneficial as expected. Therefore, cetuximab has been coupled with different radioisotopes for imaging or treatment of various tumor models in individualized therapeutic strategies such as RIT [5]. RIT with cetuximab is a feasible treatment option with a low overall incidence of systemic side effects and a favorable toxicity profile [12].

Several β^−^-particle-emitting radioisotopes are commonly used in RIT. Scandium-47 (^47^Sc) has recently been proposed as an alternative radioisotope to Lutetium-177 (^177^Lu). ^47^Sc is a low-energy β^*−*^emitter (Eβ_av_^−^ = 162 keV) with a 3.35-day half-life and shows a primary γ-ray at 159 keV, which is suitable for therapeutic applications and single-photon emission computed tomography (SPECT) imaging [13, 14]. ^47^Sc is a trivalent radioisotope that can be readily conjugated to peptides and proteins using bifunctional chelating agents (BFCAs) [15], which have a reactive portion for covalent binding to proteins and another portion capable of strongly binding to the radiometal (radioisotope), resulting in a physiologically stable complex. Additionally, ^47^Sc displays favorable physical properties and chemistry for conjugation to monoclonal antibody chelate systems [16]. Since it is chemically similar to ^90^Y and close to ^177^Lu, the same ligands developed for ^90^Y or ^177^Lu can be used to chelate ^47^Sc [17]. The properties of ^47^Sc confer some key advantages over ^177^Lu, which is used in undergoing clinical trials. ^47^Sc has a shorter half-life than ^177^Lu owing to its rapid pharmacokinetics. Therefore, it may be useful for the treatment and diagnosis of tumors [18]. However, the therapeutic efficacy of ^47^Sc in preclinical and clinical studies is still largely unknown compared with that of ^177^Lu.

Although RIT has yielded clinical benefits, intrinsic and acquired resistance are common outcomes. The mechanisms of resistance refer to intrinsic and extrinsic alterations in tumors. Resistance to targeted therapy impairs its clinical use and efficiency. Therefore, to resolve the issue of resistance to RIT, such as cetuximab conjugated with therapeutic radioisotopes, it is necessary to understand the underlying molecular mechanisms and regulatory genes. This study investigated the downregulation of the *RUNX3* (Runt-related transcription factor 3) gene expression in NSCLC cell lines with abnormally high EGFR expression. RUNX3, a member of the Runt family of transcription factors, plays an important role in developmental and biological processes. It is a well-recognized tumor suppressor in various tumors and is dysregulated in cancer [19]. RUNX3 is linked to multiple stages of the DNA damage response, namely damage sensing, and repair [20]. RUNX3 is frequently inactivated in human cancer cell lines and cancer samples through hemizygous deletion, promoter hypermethylation, histone modification, and protein mislocalization [21]. The inactivation of RUNX3 contributes to genome instability [22] and promotes resistance to radiotherapy [23]. However, it is unknown whether RUNX3 is involved in DNA damage and repair mechanisms induced by RIT. Thus, the molecular mechanism that regulates susceptibility to EGFR-targeted RIT is still unclear.

Here, we investigated the potential therapeutic efficacy of ^47^Sc-conjugated cetuximab in EGFR-targeted therapy in NSCLC cells. This study aimed to develop ^47^Sc-based radiopharmaceuticals and understand the molecular mechanisms of resistance to RIT induced by cetuximab conjugated with ^47^Sc.

## Methods

### Cell lines and cell culture

Three human NSCLC cell lines (H520, H1975, and HCC827) were purchased from the American Type Culture Collection (ATCC; Manassas, VA, USA) and cultured in RPMI-1640 medium (Gibco BRL, Thermo Fisher Scientific, Waltham, MA, USA) supplemented with 10% fetal bovine serum (Gibco BRL) and 1% penicillin/streptomycin (Gibco BRL). All cells were incubated at 37 °C in a 5% CO_2_ atmosphere.

### Western blotting analysis

Expression of the protein of interest was assessed using western blotting. Briefly, the cells were lysed with RIPA lysis buffer (Thermo Fisher Scientific, Cat# 89,900) containing a protease inhibitor cocktail (Sigma-Aldrich, Cat# P8340), and the lysates were quantified using a BCA protein assay kit (Thermo Fisher Scientific, Cat# 23,235). Cell lysates were separated using SDS-PAGE and transferred to polyvinylidene difluoride (PVDF) membranes (Millipore, Cat# IPVH00010). Proteins were identified using individual antibodies, and the protein signals were detected using an enhanced chemiluminescence kit (Millipore, Cat# WBKLS0500). All experiments were repeated at least thrice.

### Antibodies

Antibodies targeting EGFR (Cat# 2232), phospho-EGFR (Cat# 2234), MAPK (Cat# 9102), phospho-MAPK (Cat# 9101), AKT (Cat# 9272), phospho-AKT (Cat# 9271), PARP (Cat# 9542), cleaved PARP (Cat# 5625), caspase-3 (Cat# 14,220), cleaved caspase-3 (Cat# 9664), phospho-histone H2A.X (Cat# 9718), phospho-DNA–protein kinase catalytic subunit (PKcs) (Cat# 68,716), Bax (Cat# 2772), and beta-tubulin (Cat# 2146) were obtained from Cell Signaling Technology (Danvers, MA, USA). Antibodies targeting p53 (Cat# sc-126), DNA-PKcs (Cat# sc-5282), Ku70 (Cat# sc-5309), and p27 (Cat# sc-1641) were obtained from Santa Cruz Biotechnology (Dallas, TX, USA). The Antibody targeting RUNX3 (Cat# ab40278) was purchased from Abcam (Cambridge, MA, USA). All antibodies were diluted 1:1000.

### Reagents

Cetuximab (Cat# HY-P9905) was purchased from MedChemExpress (Monmouth, NJ, USA). The human IgG1 isotype (Cat# BE0297) was purchased from BioXCell (Lebanon, NH, USA) and used as a control. The jetOPTIMUS transfection reagent for transient transfection in cells was purchased from Polyplus (Alsace, Grand Est, France). The bifunctional chelating agent [(R)-2-Amino-3-(4-isothiocyanatephenyl) propyl]-trans-(S, S)-cyclohexane-1,2-diamine-pentaacetic acid (*p*-SCN-Bn-CHX-A-DTPA, Cat# B-355) was purchased from Macrocyclics Inc. (Richardson, TX, USA).

### Plasmids, DNA transfection and immunoprecipitation (IP)

The pCS4-3Myc-vector and pCS4-3Myc-RUNX3 were kindly provided by Dr. Suk-Chul Bae (Chungbuk National University, Korea). Transient transfections with the pCS4-3Myc-vector and pCS4-3Myc-RUNX3 were performed using the jetOPTIMUS transfection reagent (Polyplus, Alsace, Grand Est, France). Cell lysates were incubated with RUNX3 antibody for 12–16 h at 4 °C and then with protein A/G PLUS Agarose (Santa Cruz Biotechnology, Dallas, TX, USA) for 3 h at 4 °C. Immunoprecipitates were assessed using western blotting analysis.

### Cell growth inhibition assay

Cells (1 × 10^4^ per well) were plated into 96-well plates and incubated at 37 °C overnight. The cells were treated with 10 μg/ml cetuximab diluted in a cell culture medium. Cell viability was measured in triplicate per plate. After 72 h of treatment at 37 °C, a water-soluble tetrazolium salt (WST) solution (DOGEN, Cat# EZ-1000) was added to each well, and the reaction was allowed to proceed for 1 h at 37 °C. The absorbance of each well at 450 nm was measured and directly correlated with the number of remaining viable cells. Absorbance data were normalized to the percentage of human IgG1 isotype-treated controls and plotted.

### BrdU cell proliferation assay

Cells (1 × 10^4^ per well) were plated into 96-well plates and incubated at 37 °C overnight. The cells were treated with 10 μg/ml cetuximab and/or 0.5 μg/ml Myc-RUNX3 diluted in a cell culture medium. BrdU cell proliferation was measured in triplicate per plate according to the manufacturer’s instructions (BrdU Cell Proliferation assay, Cat # 2757, Millipore, Billerica, MA, USA). The absorbance at 450 nm was measured. All experiments were performed with triplicate samples.

### Apoptosis assay

Cell lysates equivalent to 1 × 10^4^ per ml after 72 h of treatment with ^47^Sc-DTPA-cetuximab and/or RUNX3 were collected separately for the assay (Cell Death Detection ELISA assay; Cat #11,544,675,001; Roche, Basel, Switzerland) according to the manufacturer’s instructions. The absorbance at 490 nm was measured. All experiments were performed with triplicate samples.

### Production of ^47^Sc

^47^Sc was produced by the irradiation of the CaCO_3_ target at the HANARO research reactor in KAERI (Korea Atomic Energy Research Institute). The enriched ^46^Ca (5.2% in ^46^Ca) target was irradiated at the IP-15 hole (30 MW, thermal neutron flux: 1.4 × 10^14^ n/cm^2^/s), which has the highest neutron flux among the on-power loading irradiation holes of HANARO, for 7 days. ^47^Sc was prepared by chromatographic separation of the irradiated target. The cells were treated with 74 kBq ^47^Sc per ml of cell culture medium.

### Radiolabeling

Cetuximab (1 mg/ml) was incubated with a tenfold molar excess of *p*-SCN-Bn-CHX-A-DTPA in 0.01 M borate buffer (pH 8.2) for 3 h at room temperature. The conjugated antibodies were then purified. The *p*-SCN-Bn-CHX-A-DTPA-conjugated cetuximab was labeled with 7.4 MBq ^47^Sc in 0.05 M ammonium acetate buffer (pH 6) for 0.5–1 h at room temperature.

### Radiochemical purity

Radiochemical purity was determined using instant thin-layer chromatography (ITLC). ITLC of the purified radioimmunoconjugate was performed using silica gel strips (ITLC-SG; Agilent Technologies, CA, USA) with 0.1 M sodium citrate or 1 M ammonium acetate/methanol, 50/50 (v/v) as the mobile phase. The radiochemical purity and retention factor (Rf) were assessed by calculating the area under the curve using the WinScan software by Eckert and Ziegler.

### In vitro serum stability

The in vitro serum stability of ^47^Sc-DTPA-cetuximab was analyzed in human serum, saline, and 10% RPMI-1640 (cell culture medium). A 1:5 mixture of ^47^Sc-DTPA-cetuximab in human serum, saline, and 10% RPMI-1640 was incubated at 37 °C for 7 days. On selected days (days 1, 2, 3, and 7), the stability of ^47^Sc-DTPA-cetuximab was confirmed using ITLC-SG. All experiments were performed with triplicate samples.

### Immunoreactivity

The immunoreactivity of ^47^Sc-DTPA-cetuximab was evaluated using the Lindmo method and an in vitro cell-binding assay. Following the Lindmo method, cells (1–5 × 10^6^) were incubated with 0.185 MBq ^47^Sc-DTPA-cetuximab for 1 h at 4 °C. The cells were washed thrice with cold PBS and centrifuged at 1000 rpm for 5 min. Radioactivity was quantified using a Wallac 1470 automated gamma counter (PerkinElmer Life Sciences). For the in vitro cell-binding assay, cells (1 × 10^6^) were incubated with 0.185 MBq ^47^Sc-DTPA-cetuximab for 1 h at 4 °C in the presence of 10 × unlabeled cetuximab and IgG as the control. The cells were washed thrice with cold PBS and centrifuged at 1000 rpm for 5 min. Radioactivity was quantified using a Wallac 1470 automated gamma counter. All experiments were performed with triplicate samples.

### Statistical analysis

Statistical analyses were performed using GraphPad Prism v7.0. The Student’s t-test or ANOVA, followed by multiple comparisons testing, were used to compare the experimental groups.

## Results

### EGFR-overexpressing NSCLC cells have diverse genetic mutations and expression levels

**Fig. 1:**
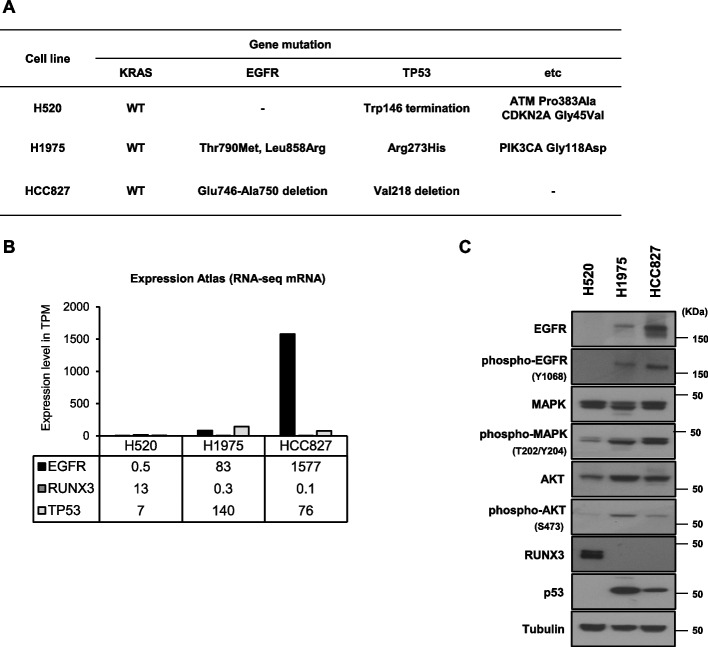
Characterization of EGFR expression in NSCLC cells. **A** Summary of the genetic mutations in the NSCLC cell lines we used. **B** The EGFR, RUNX3, and TP53 mRNA levels in the NSCLC cell lines with EGFR expression from the Expression Atlas RNA-seq data. (TPM; transcripts per kilobase million). **C** Western blotting analysis of EGFR and major downstream regulators of EGFR signaling in EGFR-expressing NSCLC cells

RNA-Seq database from the Expression Atlas (http://ebi.ac.uk/gxa) were used to obtain information on gene and protein expression in different cancer types. To investigate the function of EGFR mutations involving the tyrosine kinase domain in NSCLC, we used three NSCLC cell lines with wild-type *KRAS* and various EGFR expression levels (due to *EGFR* genetic mutations) to compare the therapeutic efficacy of cetuximab and ^47^Sc-DTPA-cetuximab. Cetuximab confers benefits against wild-type *KRAS* tumors [23]. H520 cells did not express EGFR and were used as a negative controls. H1975 and HCC827 cells harbored point mutations in *EGFR*, including deletions in exon 20 Thr790Met/exon 21 Leu858Arg and exon 19 Glu746-Ala750 deletions, respectively (Fig. 1A). In addition, the three NSCLC cell lines exhibited abnormal expression and function due to point mutations in *TP53* (Fig. 1A, B, and C). *TP53* is known as a tumor suppressor gene in various tumors, including NSCLC [24], and the *TP53* mutation is referred to as a guardian of the cancer cell [25]. EGFR-overexpressing NSCLC cell lines also exhibited suppressed *RUNX3* expression (Fig. 1B, C). *RUNX3* is a tumor suppressor that prevents lung adenocarcinoma [26], and its inactivation plays an important role in lung tumorigenesis [27]. We analyzed mRNA and protein expression levels following gene mutation and inactivation of *EGFR, TP53,* and *RUNX3* (Fig. 1B, C).

### Cetuximab induces cell cycle arrest and cell death in EGFR-overexpressing NSCLC cells

**Fig. 2:**
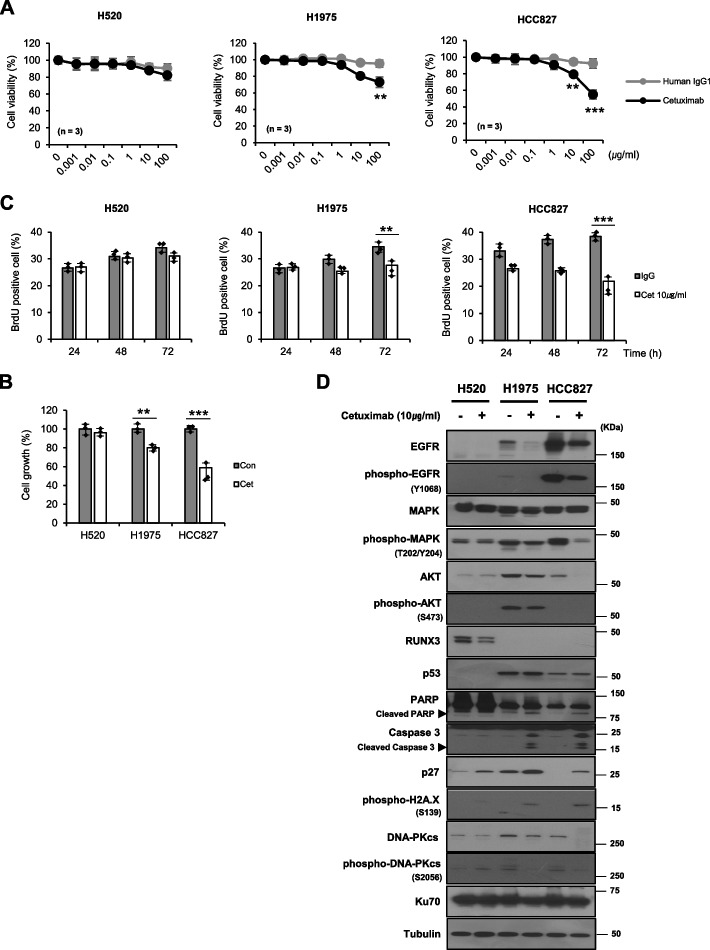
Suppressive effects of cetuximab on EGFR-overexpressing NSCLC cells. **A** The cytotoxic effect of cetuximab in EGFR-overexpressing NSCLC cells exposed to different concentrations of cetuximab. The viability of EGFR-overexpressing NSCLC cells was evaluated using a cell growth inhibition assay. Effect of cetuximab on (**B**) the growth and (**C**) proliferation of EGFR-overexpressing NSCLC cells, using cell growth inhibition and BrdU assays. **D** Western blotting analysis of the indicated proteins in EGFR-overexpressing NSCLC cells treated with cetuximab

We next assessed the inhibitory effect of cetuximab as an EGFR-targeting therapeutic agent in EGFR-overexpressing NSCLC cells. Specifically, we examined whether cetuximab reduces EGFR expression and inhibits cell growth in NSCLC cells with variable levels of EGFR expression. First, we determined the concentration of cetuximab required to inhibit cell growth. Cell viability decreased in a dose-dependent manner at concentrations of cetuximab > 10 μg/ml. In particular, cell viability was effectively reduced in HCC827 cells with high EGFR expression (Fig. 2A). Cell growth was inhibited in cells with high EGFR expression after application of 10 μg/ml of cetuximab (Fig. 2B) and the number of BrdU-positive cells decreased (Fig. 2C) compared with that after treatment with the IgG control antibody. These results suggest a correlation between the cell-binding affinity of cetuximab and the level of EGFR overexpression. Thereafter, we examined the cell growth inhibition and induction of cell death in NSCLC subjected to EGFR-targeted cetuximab treatment. In cells with high EGFR expression, cetuximab induced the activation of more cell death protein markers, such as cleaved PARP and cleaved caspase 3, than the IgG control antibody. In addition, the expression level of cell cycle arrest protein (p27) at the G_1_/S phase, DNA damage sensor protein (phosphor-histone H2A.X), and DNA repair regulator protein (DNA-PKcs and phosphor-DNA-PKcs) changed upon cetuximab treatment compared with that in the control (Fig. 2D). These results demonstrate that cetuximab shows therapeutic efficacy via cell cycle arrest at the G_1_/S phase, DNA damage, and cell death; therefore, cetuximab has potential therapeutic efficacy against EGFR-overexpressing NSCLC and can be used as a targeting vehicle and in combination schemes to improve EGFR-targeted NSCLC therapy.

### Radiolabeling of cetuximab with ^47^Sc

**Fig. 3:**
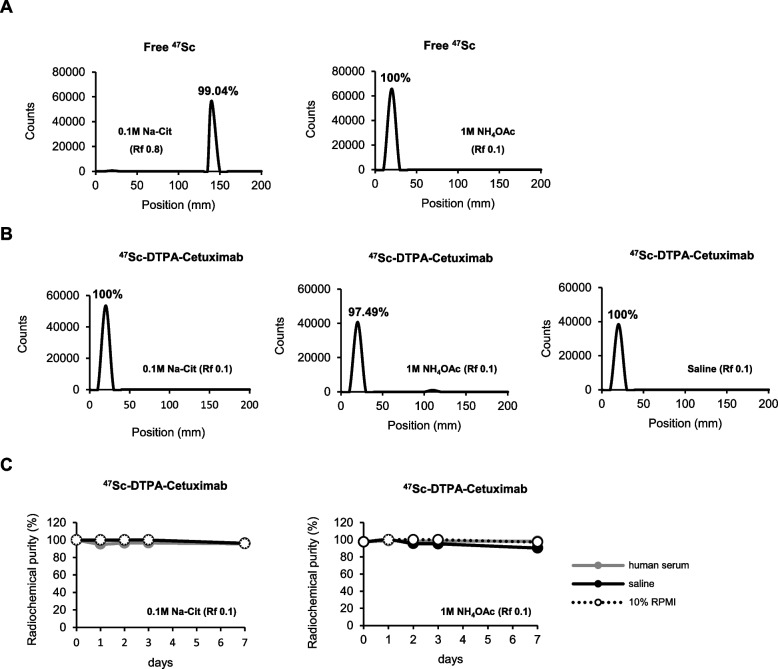
Radiolabeling of cetuximab with ^47^Sc. The radiochemical purity of (**A**) free ^47^Sc produced in the HANARO reactor and (**B**) ^47^Sc-DTPA-cetuximab was determined using instant thin-layer chromatography with silica gel strips (ITLC-SG). **C**) The radiochemical stability of the ^47^Sc-DTPA-cetuximab determined using ITLC-SG

To increase therapeutic efficacy, we radiolabeled cetuximab with the therapeutic isotope ^47^Sc. We first examined the radiochemical purity of the free ^47^Sc produced in the HANARO reactor before cetuximab radiolabeling using ITLC-SG (Fig. 3A). We then radiolabeled cetuximab with ^47^Sc via DTPA, which is a bifunctional chelating agent, and obtained a high radiolabeling yield of > 97.49% (Fig. 3B). The radiochemical purity of ^47^Sc-DTPA-cetuximab was stable (> 95%) in serum and NSCLC cell-culture media (10% RPMI-1640) in vitro within 7 days after radiolabeling (Fig. 3C).

### ^47^Sc-DTPA-cetuximab displays increased therapeutic efficacy than cetuximab alone in EGFR-overexpressing NSCLC cells

**Fig. 4:**
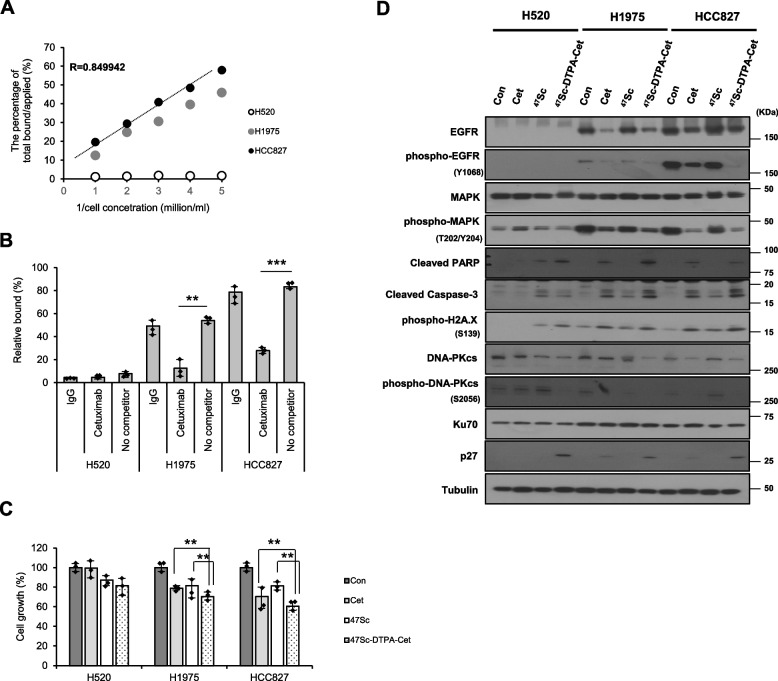
Characterization of ^47^Sc-DTPA-cetuximab and therapeutic efficacy against EGFR-overexpressing NSCLC cells. **A** Immunoreactivity of ^47^Sc-DTPA-cetuximab using the Lindmo method. **B** Binding affinity of ^47^Sc-DTPA-cetuximab using blocking assays. **C** Effect of ^47^Sc-DTPA-cetuximab on the growth of EGFR-overexpressing NSCLC cells. **D** Western blotting analysis of the indicated proteins in EGFR-overexpressing NSCLC cells treated with ^47^Sc-DTPA-cetuximab

To characterize ^47^Sc-DTPA-cetuximab, we first performed immunoreactivity and cell-binding assays using ^47^Sc-DTPA-cetuximab. Immunoreactivity, assessed using the method described by Lindmo, was close to 85%, indicating that labeling did not modify the binding of ^47^Sc-DTPA-cetuximab to the EGFR antigen (Fig. 4A). The binding affinity of ^47^Sc-DTPA-cetuximab to variably EGFR-overexpressing NSCLC cells was confirmed using competitive binding assays. ^47^Sc-DTPA-cetuximab showed a binding pattern similar to that of cetuximab, and the cellular binding of radioimmunoconjugates in EGFR-overexpressing NSCLC cells correlated with EGFR expression levels (Fig. 4B). Thereafter, we investigated whether ^47^Sc-DTPA-cetuximab exhibits increased therapeutic efficacy compared with cetuximab. ^47^Sc-DTPA-cetuximab inhibited EGFR-overexpressing NSCLC cell growth to a greater extent than either cetuximab or ^47^Sc alone (Fig. 4C). We also observed that the expression levels of cell death proteins (cleaved PARP and cleaved caspase 3) and p27 increased after treatment with ^47^Sc-DTPA-cetuximab and were similar to those after treatment with cetuximab alone. In addition, the emitted ionizing radiation (β irradiation) from ^47^Sc and ^47^Sc-DTPA-cetuximab increased the expression of phosphor-histone H2A.X compared to that in the control. The DNA-PKcs and phospho-DNA-PKcs expression levels also decreased after application of ^47^Sc-DTPA-cetuximab (Fig. 4D), suggesting that EGFR-overexpressing NSCLC cells are more sensitive to anticancer therapy following ^47^Sc-DTPA-cetuximab treatment. The results were similar to those obtained after treatment with cetuximab alone; however, radiolabeling of cetuximab with ^47^Sc was more effective in killing NSCLC cells by targeting the EGFR antigen and inducing DNA damage caused by β irradiation emitted from the conjugated ^47^Sc.

### RUNX3 regulates cetuximab-dependent DNA damage and repair process in EGFR-overexpressing NSCLC cells

**Fig. 5:**
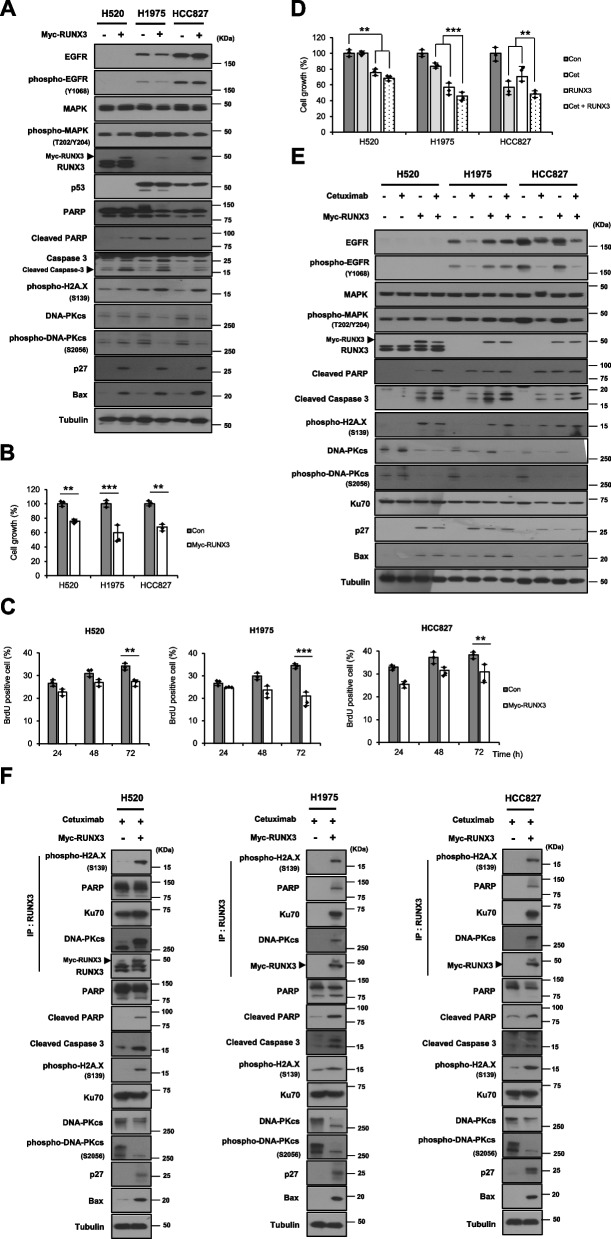
RUNX3 affects cetuximab-dependent DNA damage and repair response in EGFR-overexpressing NSCLC cells. **A** Western blotting analysis of the indicated proteins in EGFR-overexpressing NSCLC cells transfected with RUNX3 transfection. Effect of RUNX3 expression on (**B**) the growth and (**C**) proliferation of EGFR-overexpressing NSCLC cells, using cell growth inhibition and BrdU assays. **D** Effect of the combination of RUNX3 expression and cetuximab on the growth of EGFR-overexpressing NSCLC cells. **E** Western blotting analysis of the indicated proteins in EGFR-overexpressing NSCLC cells treated with cetuximab and transfected RUNX3. **F** Following cetuximab-induced DNA damage and repair, RUNX3 interacts with DNA damage and repair response-related proteins and induces cell death-related protein expression in the presence of RUNX3. Cell lysates were used for an immunoprecipitation assay using an anti-RUNX3 antibody

We observed that cetuximab affects phospho-histone H2A.X activity and DNA repair regulator activity (DNA-PKcs and phospho-DNA-PKcs). We then raised the question of which gene is involved in cetuximab-dependent DNA damage and repair. We verified that RUNX3 gene expression was downregulated in NSCLC cells with high EGFR expression via the RNA-Seq database search and western blotting analysis (Fig. 1). To investigate the function of RUNX3 expression in EGFR-overexpressing NSCLC cells, we examined whether RUNX3 re-expression was related to cetuximab-dependent cell death. We observed that RUNX3 re-expression increased the expression levels of cell death proteins (cleaved PARP and cleaved caspase 3), p27, and phospho-histone H2A.X. In addition, we observed that DNA repair regulatory activity (DNA-PKcs and phospho-DNA-PKcs**)** decreased by RUNX3 re-expression. Increased RUNX3 expression also induced the expression of Bax, which is a major regulator of programmed cell death and a known downstream target of RUNX3 during cell death [28] (Fig. 5A). Transient transfection of RUNX3 in EGFR-overexpressing NSCLC cells significantly inhibited cell growth (Fig. 5B) and decreased the number of BrdU-positive cells (Fig. 5C) compared with those in the empty vector control. These findings indicated that RUNX3 re-expression in NSCLC cells with downregulated RUNX3 expression mediated susceptibility to cetuximab. Similar results were observed in the anticancer study on the efficacy of EGFR-targeting cetuximab in NSCLC cells (Fig. 2). Meanwhile, the transient expression of Myc-RUNX3 in H520 cells considerably induced cell growth inhibition and apoptotic cell death compared to the expression of endogenous RUNX3 (Fig. 5A, B and C). The H520 cells carry gene mutations, including *TP53* (translated p53) and *CDKN2A* (translated p14 ARF and p16 INK4A), which plays an important roles as RUNX3 target genes and co-activators of tumor suppression. Normal expression of RUNX3 in H520 cells did not function as a tumor suppressor in this study. Therefore, even if RUNX3 expression is normal, the expected cancer-suppressive effect of endogenous RUNX3 is not evident in the presence of deletions or mutations in the target genes or co-activator of RUNX3. It is expected that exogenous RUNX3 (Myc-RUNX3) can promote the cancer-suppressive effect of endogenously expressed RUNX3 via independent signal transduction of the ARF-p53 pathway. We suggest that exogenous expression of RUNX3 (Myc-RUNX3) is a substitute for defense mechanisms against cancer, in accordance with the inactivation of the ARF-p53 pathway due to gene deletions or mutations.

We then investigated whether cetuximab efficacy correlated with RUNX3 re-expression. Combined treatment with cetuximab and RUNX3 re-expression significantly suppressed the growth of NSCLC cells compared with treatment with cetuximab and RUNX3 re-expression alone (Fig. 5D). As expected, the levels of relevant proteins involved in cell death, cell cycle arrest, and DNA damage and repair were altered by combination treatment with cetuximab and RUNX3 re-expression (Fig. 5E). These results indicate that RUNX3 re-expression (RUNX3 activation) enhances susceptibility to cetuximab, suggesting that the inhibitory effect of cetuximab may be regulated by RUNX3. Therefore, we investigated the regulatory mechanism of RUNX3. We focused on the DNA damage and repair process to evaluate cetuximab efficacy. An immunoprecipitation assay was performed using RUNX3 re-expressing cells. Following cetuximab-dependent DNA damage and repair, RUNX3 and DNA damage and repair-process proteins (phospho-histone H2A.X, PARP, Ku70, and DNA-PKcs) interacted and induced cell death (cleaved PARP, cleaved caspase 3, and Bax) in the presence of RUNX3 (Fig. 5F). These results show that RUNX3 leads to cetuximab-dependent DNA damage and repair as a cell death regulator; thus, RUNX3 expression (RUNX3 activation) is likely required for effective cetuximab action in response to DNA damage and repair in EGFR-overexpressing NSCLC cells. In summary, RUNX3 plays a role in the regulation of cetuximab-dependent DNA damage sensing and inappropriate DNA repair processes (non-homologous end-joining pathway [NHEJ]) in NSCLC cells by interacting with protein linked to DNA damage and repair.

### Activation of RUNX3 enhances susceptibility against EGFR-targeted NSCLC therapy using ^47^Sc-DTPA-cetuximab

**Fig. 6:**
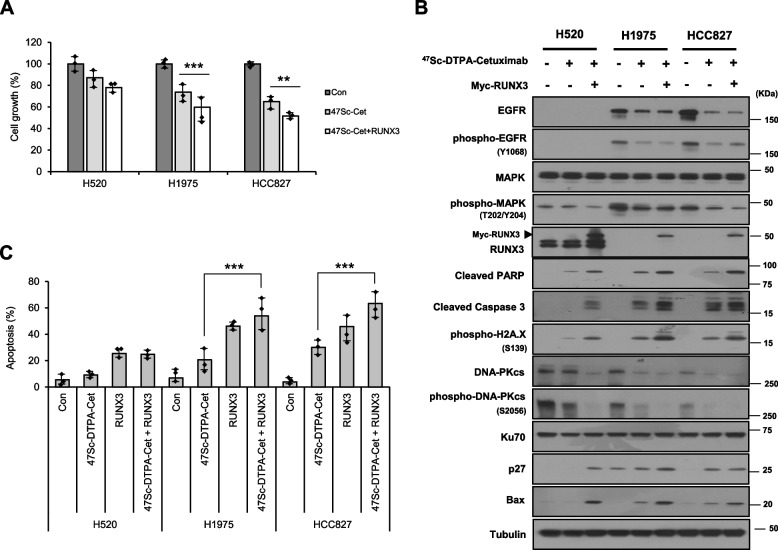
RUNX3 enhances the therapeutic efficacy of ^47^Sc-DTPA-cetuximab in EGFR-overexpressing NSCLC cells. **A** Effect of the combination of RUNX3 expression and ^47^Sc-DTPA-cetuximab on the growth of EGFR-overexpressing NSCLC cells. **B** Western blotting analysis of the indicated proteins in EGFR-overexpressing NSCLC cells treated with ^47^Sc-DTPA-cetuximab and transfected RUNX3. **C** Effect of the combination of RUNX3 expression and ^47^Sc-DTPA-cetuximab on the apoptosis of EGFR-overexpressing NSCLC cells

To further investigate RUNX3-mediated cetuximab-dependent cell death in NSCLC cells, we examined whether RUNX3 re-expression (activation) was beneficial in treatments with ^47^Sc-DTPA-cetuximab. The therapeutic effect of ^47^Sc-DTPA-cetuximab on cancer cell growth was effectively enhanced by RUNX3 expression (Fig. 6A). We also observed that RUNX3 re-expression promoted the expression of cell death proteins (cleaved PARP, cleaved caspase 3, and Bax), a cell cycle arrest protein (p27), and DNA damage sensor protein (phospho-histone H2A.X). In addition, the expression levels of DNA-PKcs and phospho-DNA-PKcs were significantly repressed when a combination of RUNX3 re-expression and ^47^Sc-DTPA-cetuximab was applied (Fig. 6B); this results suggests that RUNX3 acts as a major factor in the process of DNA damage and repair induced by cetuximab and the ionizing radiation emitting therapeutic radioisotope ^47^Sc, at least in these EGFR-overexpressing NSCLC cells. Furthermore, the combination of RUNX3 re-expression and ^47^Sc-DTPA-cetuximab considerably promoted apoptosis in the EGFR-overexpressed NSCLC cells (Fig. 6C). These results suggest that RUNX3 expression (activation) correlates with resistance to anticancer therapies, such as the combination of cetuximab and ^47^Sc.

## Discussion

**Fig. 7:**
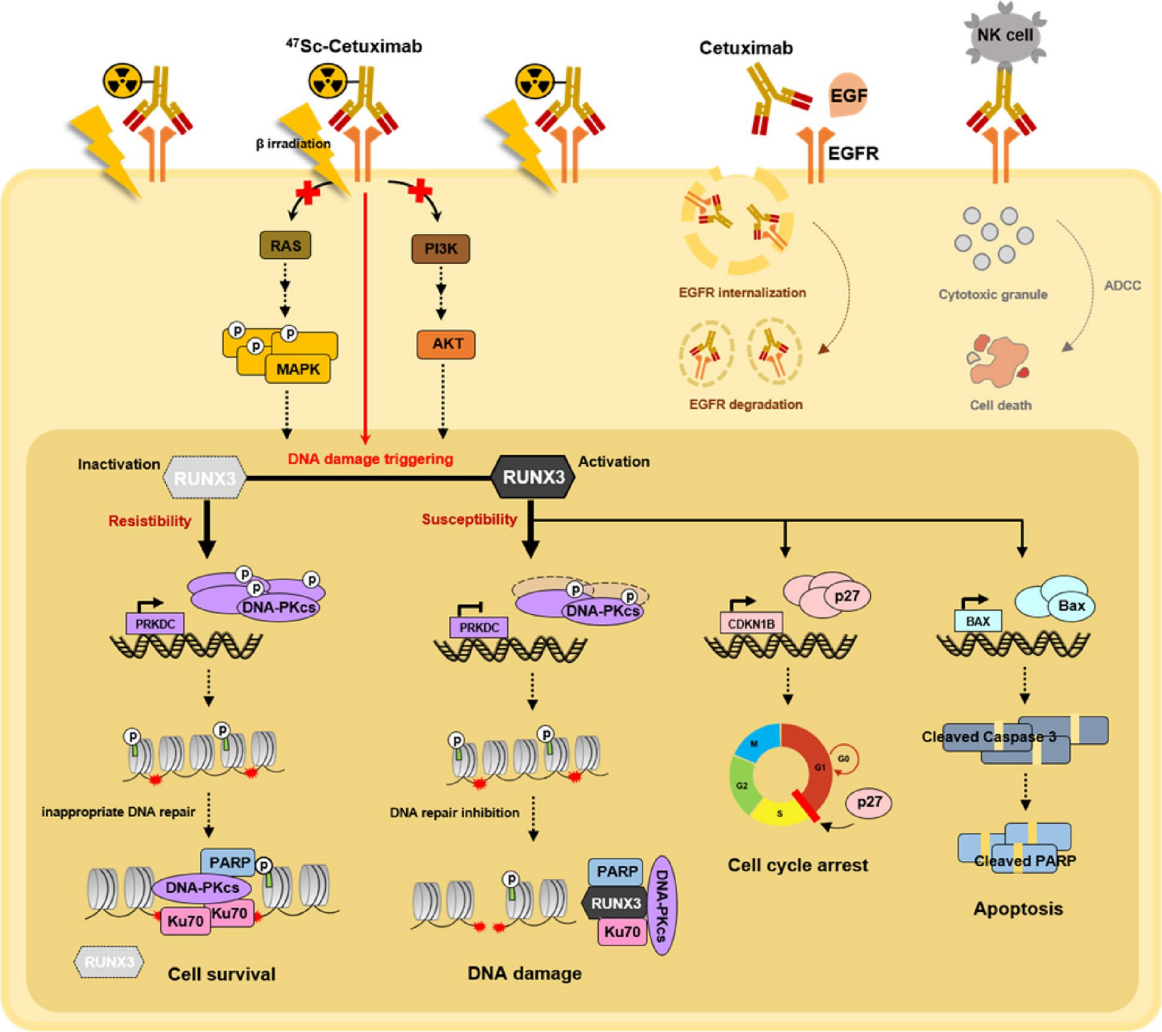
RUNX3 regulates susceptibility against EGFR-targeted NSCLC therapy using ^47^Sc-conjugated cetuximab. ^47^Sc-DTPA-cetuximab inhibits the RAS-MAPK and PI3K-AKT pathways. Activation of RUNX3 induces cell cycle arrest, apoptosis, and DNA damage accumulation. The inactivation of RUNX3 promotes an inappropriate DNA repair process. During the process of DNA damage triggered by cetuximab and ionizing radiation (β irradiation), activation of RUNX3 significantly enhances the therapeutic efficacy of ^47^Sc-DTPA-cetuximab. RUNX3 regulates susceptibility against EGFR-targeted NSCLC therapy using ^47^Sc-DTPA-cetuximab via interactions with DNA damage and repair components

The abnormal elevated expression and activity of EGFR in NSCLC are frequently associated with poor prognosis, increased tumor growth, metastasis, and resistance to various cancer therapies. As an EGFR-targeted therapy, RIT with therapeutic radioisotopes-conjugated cetuximab is frequently used in clinical and preclinical studies because of its high therapeutic efficacy. The present study identified the potential therapeutic efficacy of cetuximab and ^47^Sc via ^47^Sc-conjugated cetuximab in EGFR-overexpressing NSCLC cells. Importantly, RUNX3 expression (RUNX3 activation) was associated with resistance to cetuximab and ^47^Sc-conjugated cetuximab in these cells, indicating that RUNX3 plays an important role in cell death induced by cetuximab and ^47^Sc-conjugated cetuximab by regulating DNA damage and repair, cell cycle, and apoptosis (Fig. 7).

Cetuximab is a chimeric monoclonal antibody, one of the first anti-EGFR antibodies to be developed, and has been approved for the treatment of colorectal and head and neck squamous cell carcinoma [29]. Treatment of patients with NSCLC with cetuximab has not been approved because of its marginal clinical benefits, despite statistically significant results at phase III trials [30]. Cetuximab is currently being extensively evaluated in various preclinical studies and has shown anticancer activity in NSCLC models expressing mutated EGFR^**30**^. The present study demonstrated that cetuximab suppressed EGFR-overexpressing NSCLC cells by inhibiting cell cycle progression, preventing the DNA repair response, and promoting cell apoptosis. These results suggested that cetuximab exhibits therapeutic efficacy and can be used in EGFR-targeted NSCLC therapy. Cetuximab is frequently used as a vehicle and in combination treatments to enhance the therapeutic efficacy of EGFR-targeted cancer treatment. It is well known that the conjugation of a radioisotope to cetuximab, in combination with the specific targeting properties of this antibody, may increase therapeutic efficiency [31]. Therefore, use of appropriate radioisotopes is required. Although ^177^Lu has been widely used in cancer-targeted radioimmunotherapy, true theranostic applications with ^177^Lu are not feasible. ^47^Sc can be used in theranostic approaches in tandem with ^44^Sc or ^43^Sc as diagnostic matches [32, 33]. This goal can be achieved using ^47^Sc-based therapeutic radiopharmaceuticals for both diagnosis and therapy. However, only a few studies have described the efficacy and potential clinical application of ^47^Sc in therapeutic radiopharmaceuticals owing to limitations on export and import due to their short half-life and a lack of widely standardized production methods. In this study, we demonstrate that the self-produced ^47^Sc in the HANARO reactor has potential therapeutic efficacy and quality potential suitable for preclinical applications. In addition, the therapeutic efficacy and properties of ^47^Sc are similar to those of ^177^Lu, suggesting the possibility of developing ^47^Sc as a new radiopharmaceutical to replace ^177^Lu.

We revealed that ^47^Sc-conjugated cetuximab has increased therapeutic efficacy over cetuximab alone in inducing a DNA damage response in EGFR-overexpressing NSCLC cells. We focused on the DNA damage and repair process as the inhibitory mechanism of ^47^Sc-conjugated cetuximab in these cells. DNA double-strand breaks (DSBs) are the most serious and dangerous type of DNA damage that affects the stability of the genome and cell fate [34, 35]. In particular, the NHEJ pathway is the predominant pathway for DSBs after exposure to ionizing radiation and it is responsible for the rapid repair of up to 85% of DSBs [36]. In the NHEJ pathway, DNA-PK is important for the precise regulation of the formation and dynamics of its functional complex and for guarding genomic stability [37]. We observed that DNA-PKcs expression was reduced after treatment with both cetuximab and ^47^Sc-conjugated cetuximab in EGFR-overexpressing NSCLC cells. Thus, cetuximab and ^47^Sc-conjugated cetuximab promote genetic instability by inhibiting inappropriate DNA repair processes through the suppression of DNA-PK activity, leading to cell death in EGFR-overexpressing NSCLC cells. We conjecture that a transcription factor regulates cell death induced by cetuximab and ^47^Sc-conjugated cetuximab through the suppression of DNA-PK activity.

*RUNX3* is a well-known tumor suppressor and prognostic biomarker for patients with NSCLC [38]. RUNX3 expression is important for NSCLC development and clinical outcomes. We confirmed that RUNX3 expression was suppressed in EGFR-overexpressing NSCLC cells. Re-expression of RUNX3 (activation) induced anti-cancer effects and regulated susceptibility to cetuximab and ^47^Sc-conjugated cetuximab, highlighting the key role of RUNX3 in these processes. Furthermore, we verified that RUNX3 controls DNA damage and repair and DNA-PK activity by interacting with DNA-PKcs. RUNX3 interacts with DNA damage sensors (phospho-histone H2A.X) and DNA repair regulators (PARP and Ku70), particularly in the NHEJ pathway. The DNA-PK activity has been associated with carcinogenesis and resistance to targeted cancer therapy [39]. These findings suggest that RUNX3 regulates DNA-PK activity in response to inappropriate DNA damage and repair processes in a cancer cells or cancer cell death. Therefore, RUNX3 may be a potential regulator of inappropriate DNA repair (NHEJ pathway).

Although RIT has shown excellent therapeutic effects against hematological cancer and lymphoma, its application in solid cancers has been clinically limited due to barriers created by the tumor microenvironment. Intrinsic and acquired resistance to cancer-targeted therapies represents the central therapeutic challenge in oncology today. Resistance develops through numerous mechanisms, including increased expression of cellular drug efflux pumps, mutation of therapeutic targets, increased activity of DNA repair mechanisms, and altered expression of genes involved in apoptotic pathways [40–45]. Potential reasons for resistance to RIT with cetuximab include EGFR mutants in the tyrosine kinase domain and oncogenes *KRAS, NRAS*, or *BRAF*, which can activate EGFR downstream signaling pathways even during EGFR inhibition, and the major tumor suppressor *TP53* mutants. We examined two NSCLC cell lines (H1795 and HCC827) with different EGFR expression levels and *EGFR* gene mutations to compare the therapeutic efficacy of cetuximab and ^47^Sc-conjugated cetuximab. We could not confirm any difference in therapeutic efficacy depending on specific *EGFR* mutations. In the present study, the different EGFR mutants were not considered resistant to cetuximab or ^47^Sc-conjugated cetuximab. However, we demonstrated that RUNX3 re-expression (activation) regulated susceptibility to cetuximab and ^47^Sc-conjugated cetuximab. These results suggest that RUNX3 activity is an important regulator of sensitivity to resistance in RIT as well as in various cancer therapies. As a combination treatments are the preferred options for most cancers, a combination of therapeutic drugs with RUNX3 activity is essential to increase therapeutic efficacy.

## Conclusions

We described the therapeutic efficacy of ^47^Sc and its potential for the development of ^47^Sc-based radiopharmaceuticals against EGFR-overexpressing NSCLC. Furthermore, we found that RUNX3 is an essential regulator of susceptibility to cetuximab and ^47^Sc-conjugated cetuximab via processes related to DNA damage and repair. These results provide a basis for the development of novel radiopharmaceuticals and contribute to our understanding of the molecular mechanisms underlying DNA damage and repair that confer resistance to RIT in EGFR-overexpressing NSCLC. By proposing a mechanism to overcome resistance to caner-targeting therapies, this study offers insight into RIT resistance.

## Supplementary Information

**Additional file 1. **

## Data Availability

All data generated or analyzed during this study are included in this published article.

## Abbreviations

EGFR: Epidermal growth factor receptor
NSCLC: Non-small cell lung cancer
RIT: Radioimmunotherapy
HNSCC: Head and neck squamous cell carcinoma
CRC: Colorectal cancer
ADCC: Antibody-dependent cellular cytotoxicity
^47^Sc: Scandium-47
^1^^77^Lu: Lutetium-177
^90^Y: Yttrium-90
SPECT: Single-photon emission computed tomography
PET: Positron emission tomography
RUNX3: Runt-related transcription factor
ITLC: Instant thin-layer chromatography
SG: Silica gel
DTPA: Diethylenetriamine pentaacetate
DSB: DNA double-strand break
NHEJ: Non-homologous end-joining
DNA-PKcs: DNA-dependent protein kinase catalytic subunit
