# Supplementary material for: RUNX3 regulates the susceptibility against EGFR-targeted non-small cell lung cancer therapy using 47Sc-conjugated cetuximab
Source: BMC Cancer. 2023 Jul 12;23:652. doi: 10.1186/s12885-023-11161-1 (PMC10337176; doi:10.1186/s12885-023-11161-1)
Supplement: Supplementary file 1 — Additional file 1. [file 12885_2023_11161_MOESM1_ESM.pptx]

## Slide 1
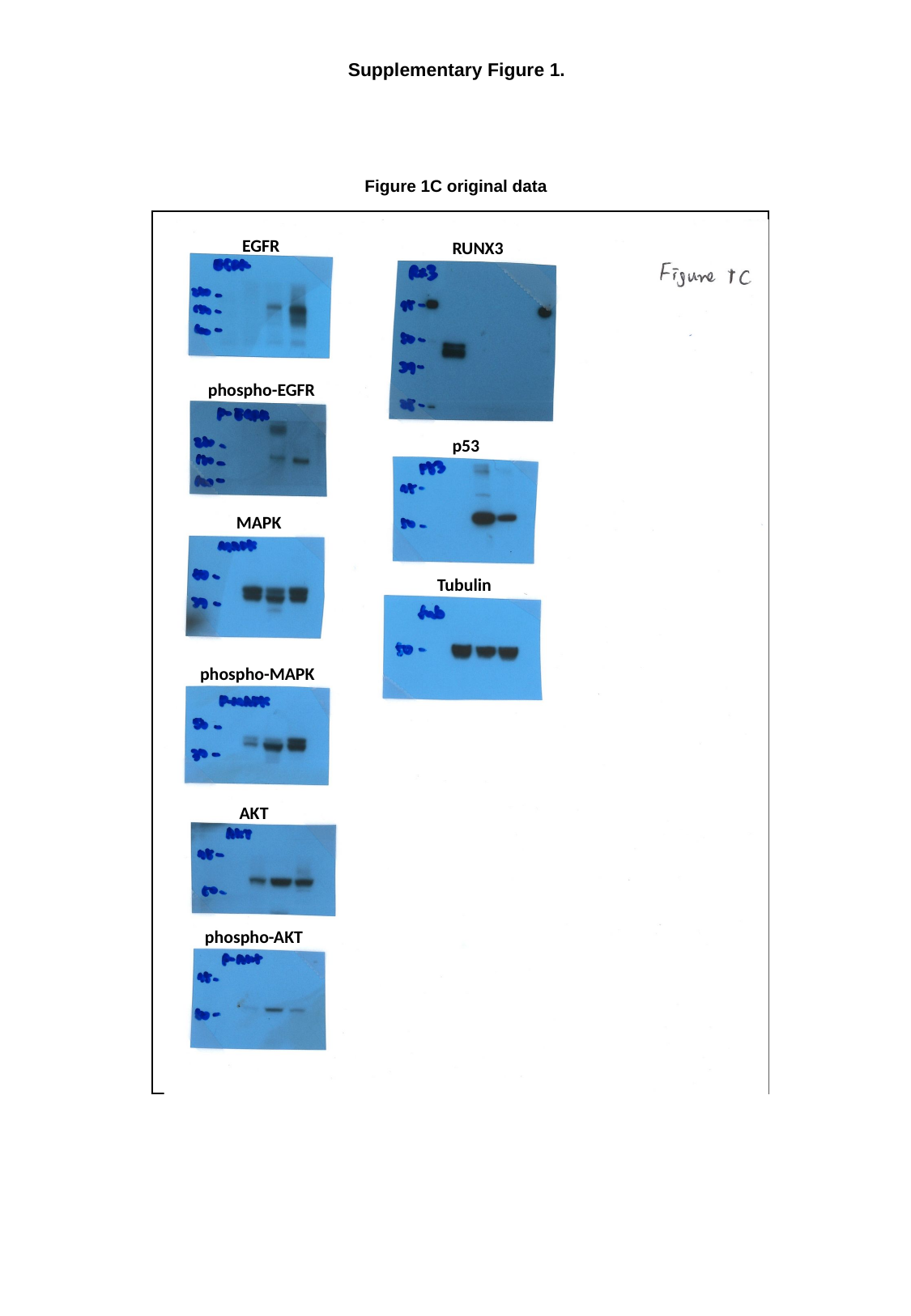

Supplementary Figure 1.
Figure 1C original data
EGFR
RUNX3
phospho-EGFR
p53
MAPK
Tubulin
phospho-MAPK
AKT
phospho-AKT

## Slide 2
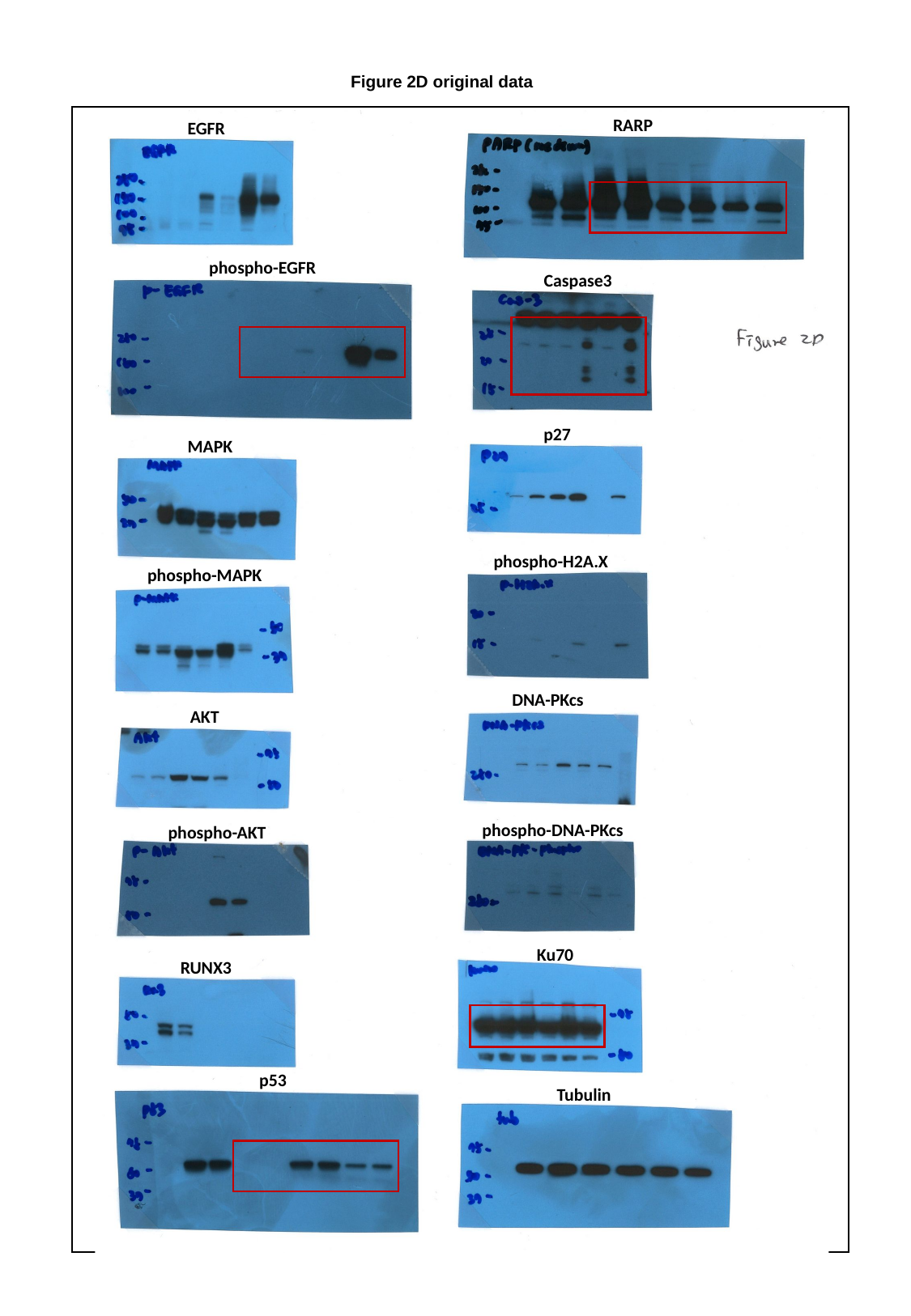

Figure 2D original data
RARP
EGFR
phospho-EGFR
Caspase3
p27
MAPK
phospho-H2A.X
phospho-MAPK
DNA-PKcs
AKT
phospho-DNA-PKcs
phospho-AKT
Ku70
RUNX3
p53
Tubulin

## Slide 3
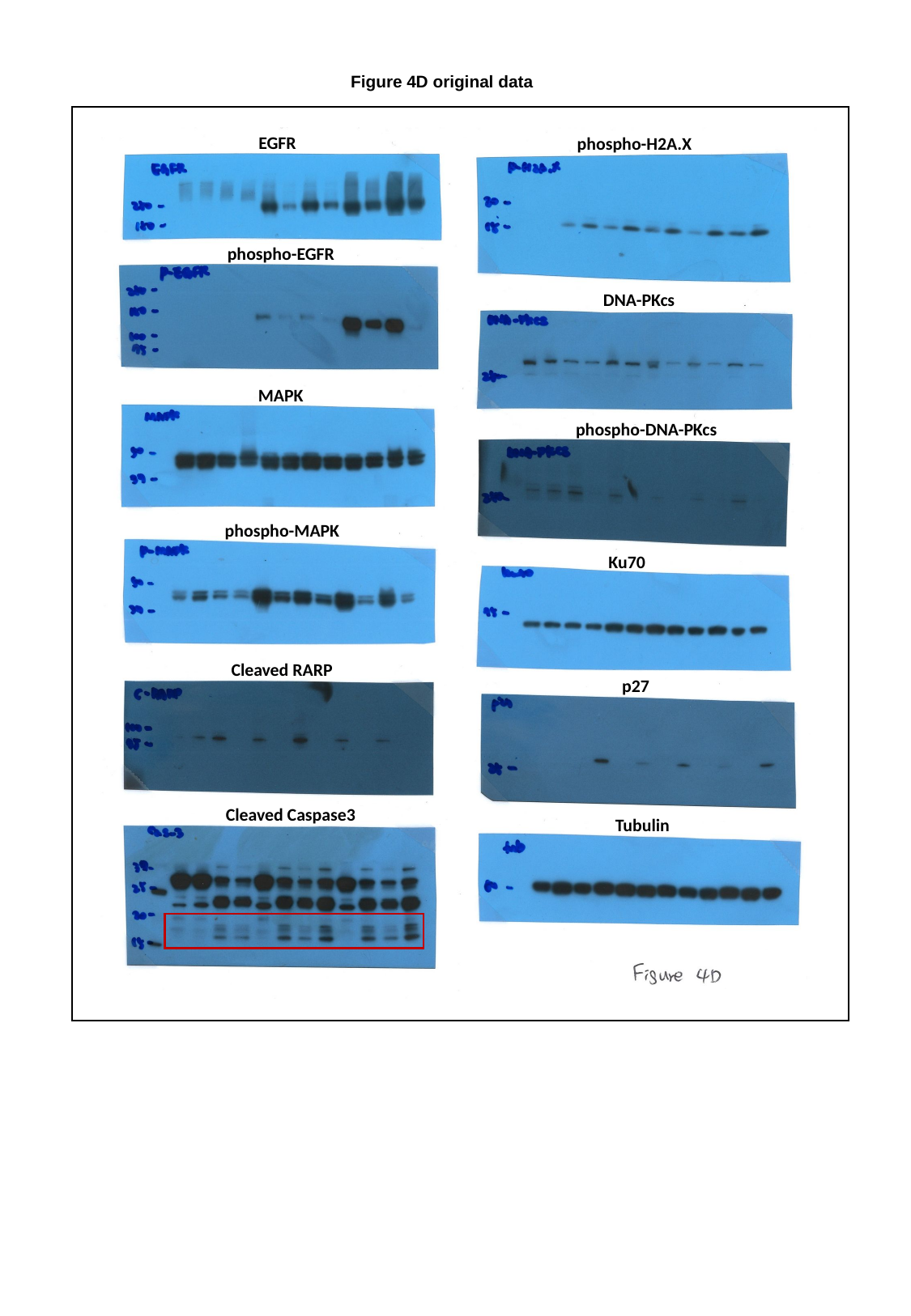

Figure 4D original data
EGFR
phospho-H2A.X
phospho-EGFR
DNA-PKcs
MAPK
phospho-DNA-PKcs
phospho-MAPK
Ku70
Cleaved RARP
p27
Cleaved Caspase3
Tubulin

## Slide 4
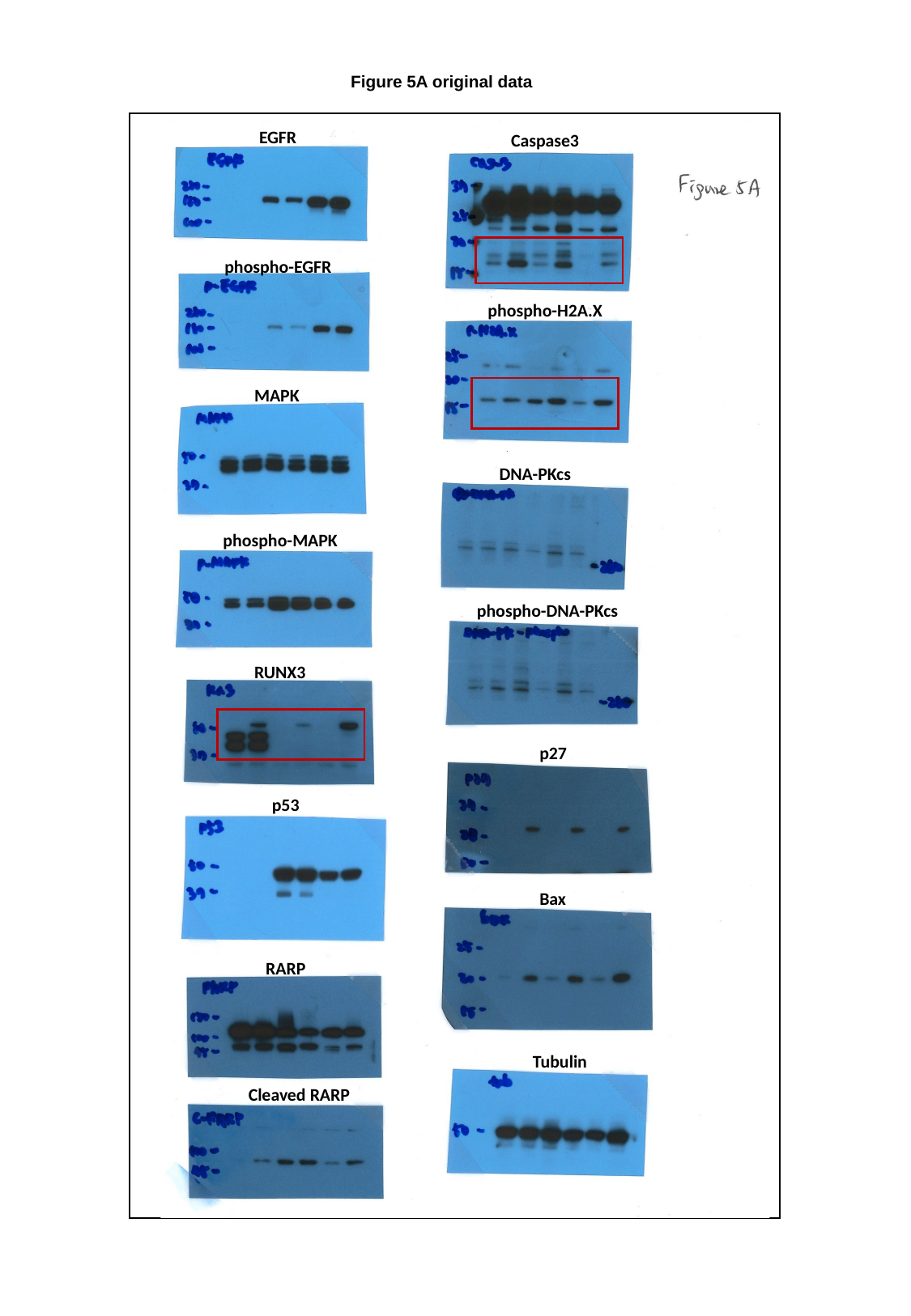

Figure 5A original data
EGFR
Caspase3
phospho-EGFR
phospho-H2A.X
MAPK
DNA-PKcs
phospho-MAPK
phospho-DNA-PKcs
RUNX3
p27
p53
Bax
RARP
Tubulin
Cleaved RARP

## Slide 5
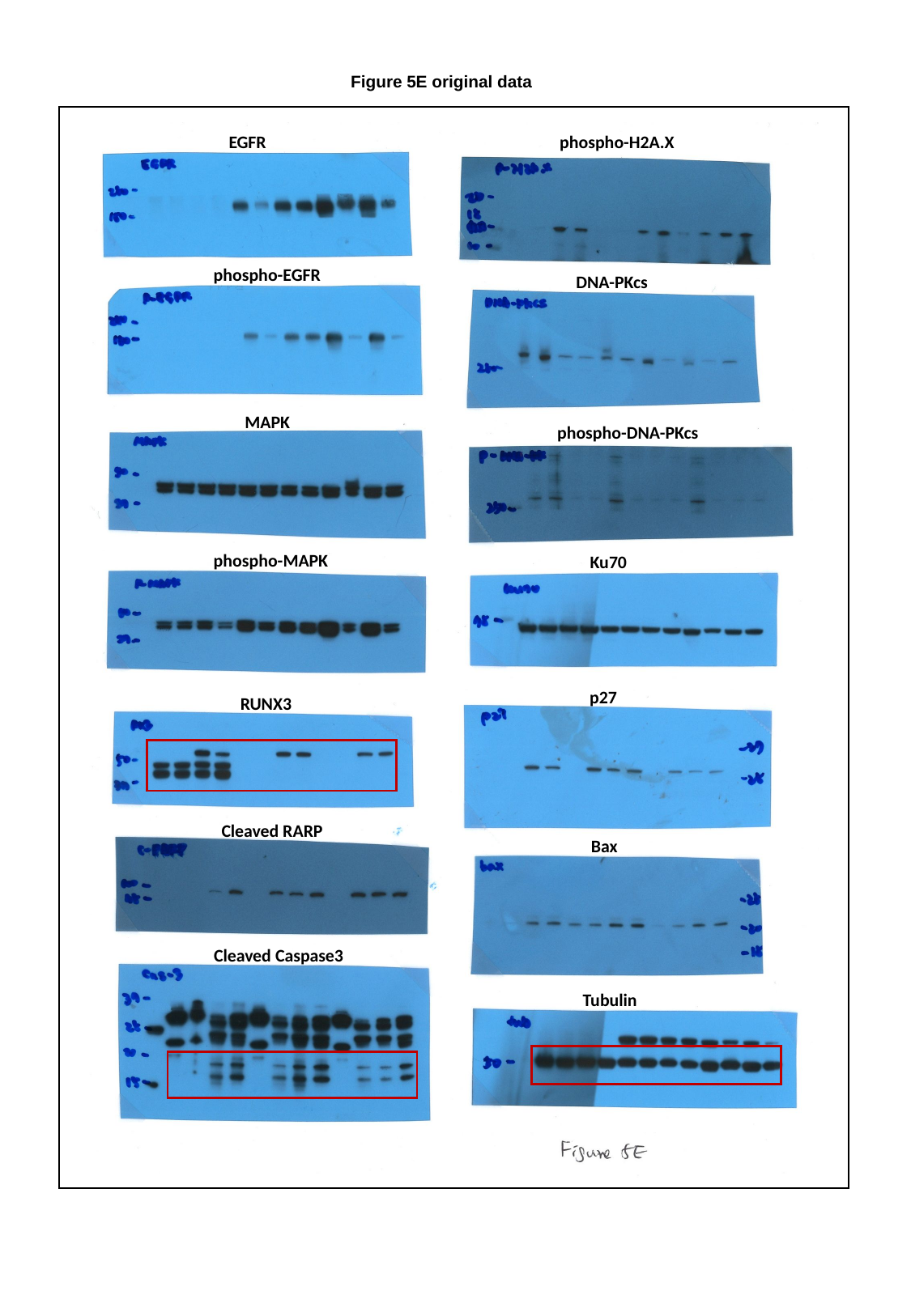

Figure 5E original data
EGFR
phospho-H2A.X
phospho-EGFR
DNA-PKcs
MAPK
phospho-DNA-PKcs
phospho-MAPK
Ku70
p27
RUNX3
Cleaved RARP
Bax
Cleaved Caspase3
Tubulin

## Slide 6
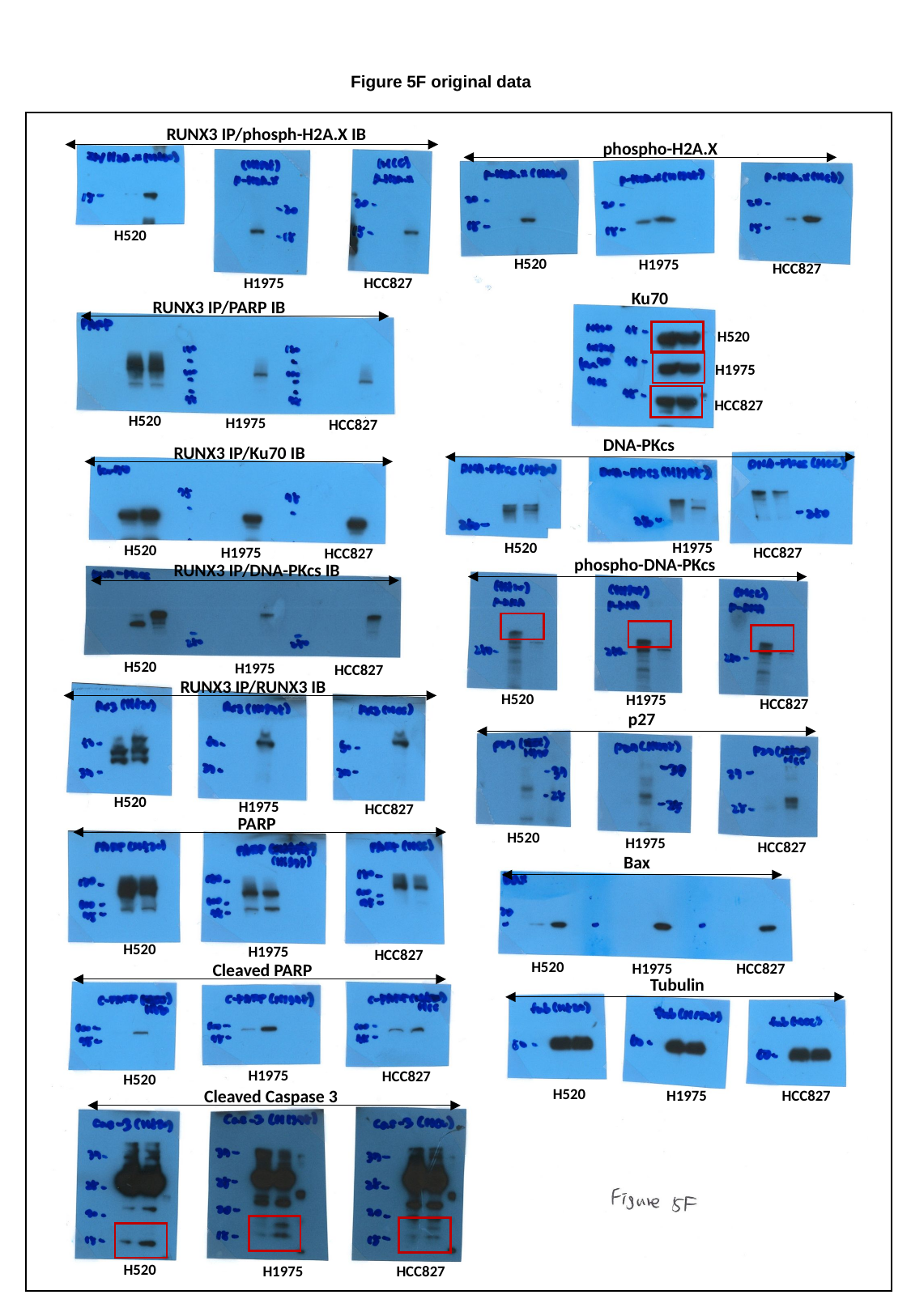

Figure 5F original data
RUNX3 IP/phosph-H2A.X IB
phospho-H2A.X
H520
H520
H1975
HCC827
H1975
HCC827
Ku70
RUNX3 IP/PARP IB
H520
H1975
HCC827
H520
H1975
HCC827
DNA-PKcs
RUNX3 IP/Ku70 IB
H520
H1975
H520
H1975
HCC827
HCC827
phospho-DNA-PKcs
RUNX3 IP/DNA-PKcs IB
H520
H1975
HCC827
RUNX3 IP/RUNX3 IB
H520
H1975
HCC827
p27
H520
H1975
HCC827
PARP
H520
H1975
HCC827
Bax
H520
H1975
HCC827
H520
Cleaved PARP
H1975
HCC827
Tubulin
H1975
HCC827
H520
H520
Cleaved Caspase 3
H1975
HCC827
H520
HCC827
H1975

## Slide 7
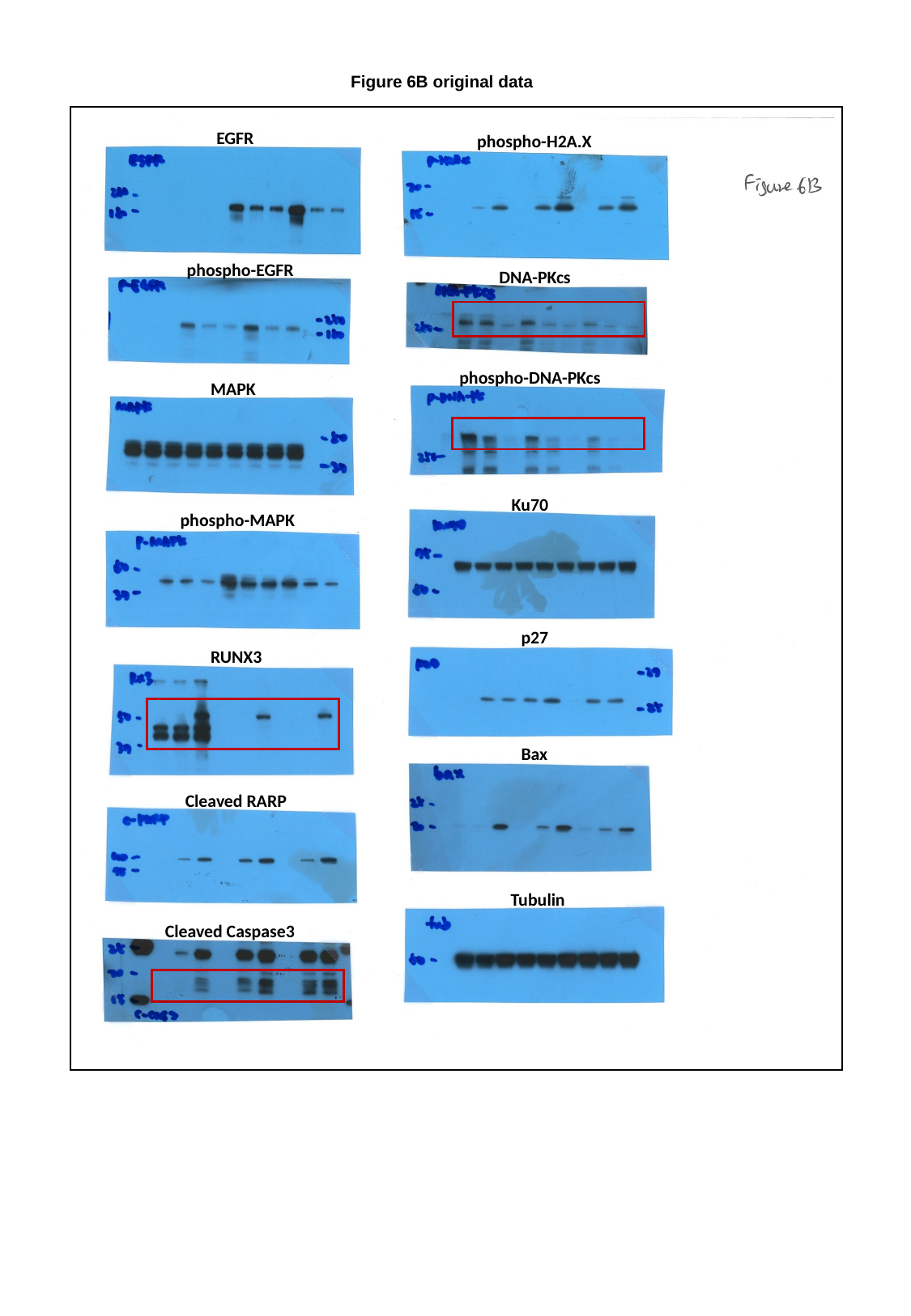

Figure 6B original data
EGFR
phospho-H2A.X
phospho-EGFR
DNA-PKcs
phospho-DNA-PKcs
MAPK
Ku70
phospho-MAPK
p27
RUNX3
Bax
Cleaved RARP
Tubulin
Cleaved Caspase3
